# Relationship of urinary glyphosate concentrations with glycosylated hemoglobin and diabetes in US adults: a cross-sectional study

**DOI:** 10.1186/s12889-024-19126-0

**Published:** 2024-06-20

**Authors:** Peng Tang, Yican Wang, Qian Liao, Yong Zhou, Huishen Huang, Jun Liang, Xiaoyun Zeng, Xiaoqiang Qiu

**Affiliations:** 1 Department of Epidemiology, School of Public Health, Guangxi Medical University, No. 22 Shuangyong Road, Nanning , Guangxi, 530021 China; 2 Department of Maternal and Child Health, School of Public Health, Peking University, Beijing, 100191 China; 3 Chinese Center for Disease Control and Prevention, National Institute for Occupational Health and Poison Control, Beijing, 100050 China; 4 School of Public Health, Xiangnan University, Chenzhou, 423000 China

**Keywords:** Diabetes, Glycosylated hemoglobin, Glyphosate, NHANES

## Abstract

**Background:**

Glyphosate is a commonly used herbicide worldwide and is purportedly associated with multiple health effects. Research assessing the association of glyphosate concentrations with glycosylated hemoglobin (HbA1c) levels and the prevalence of diabetes is scarce. We sought to evaluate the association between urinary glyphosate levels and HbA1c levels and the prevalence of diabetes.

**Methods:**

A total of 2,745 adults in the National Health and Nutrition Examination Survey from 2013 to 2016 were included in this study. Generalized linear models (GLM) were applied to evaluate the associations of glyphosate concentrations with HbA1c levels and the prevalence of diabetes. The dose–response relationship was examined using restricted cubic splines (RCS).

**Results:**

Significantly positive correlations of urinary glyphosate concentrations with HbA1c levels (percentage change: 1.45; 95% CI: 0.95, 1.96; *P* < 0.001) and the prevalence of diabetes (OR: 1.45; 95% CI: 1.24, 1.68; *P* < 0.001) were found after adjustment. Compared with the lowest quartile of glyphosate levels, the highest quartile was positively associated with HbA1c levels (percentage change: 4.19; 95% CI: 2.54, 5.85; *P* < 0.001) and the prevalence of diabetes (OR: 1.89; 95% CI: 1.37, 2.63; *P* < 0.001). The RCS curves demonstrated a monotonically increasing dose–response relationship between urinary glyphosate levels and the prevalence of diabetes and HbA1c levels.

**Conclusions:**

Urinary glyphosate concentrations are positively associated with HBA1c levels and the prevalence of diabetes. To verify our findings, additional large-scale prospective investigations are required.

**Supplementary Information:**

The online version contains supplementary material available at 10.1186/s12889-024-19126-0.

## Introduction

Glyphosate (N-(phosphonomethyl) glycine) is an organophosphorus compound that is the primary active component of glyphosate-based herbicides (GBHs). The herbicide glyphosate has a broad spectrum of activity and was introduced into agricultural production in 1974 for weed control [1]. Since the 1990s, the applications of glyphosate have increased rapidly worldwide [1]. Glyphosate is currently the most extensively used agricultural pesticide in both the United States (US) and the rest of the world [2].

Due to the inert carbon-phosphorus bond in the molecule, glyphosate strongly resistant to degradation [3]. Due to its widespread use, glyphosate is widely present in ecosystems and glyphosate residues in the environment and plants have increased. [4]. Glyphosate is the active ingredient in a large amount of broad-spectrum herbicides used in residential, commercial, and agricultural applications. The general population may be exposed to glyphosate through dermal contact with consumer products, crops, leaves, or soil containing residues of the chemical; through ingestion of plants, crops, food, or water containing residues of the chemical; or through inhalation of mists or sprays when using products containing the chemical [5]. Nearly 80% of the general U.S. population over the age of 6 had measurable levels of glyphosate in their urine, according to recent data from the National Health and Nutrition Examination Survey (NHANES) (2013–2014) [6]. In addition, another study showed that glyphosate levels were rising between 2001 and 2013 among German adults [7].

In recent years, the prevalence of diabetes has risen rapidly worldwide [8]. According to the International Diabetes Federation, as of 2021, 537 million people (aged 20–79 years) worldwide are currently living with diabetes, and that figure is expected to rise to 643 million by 2030 and 783 million by 2045, respectively [9]. The prevention and control of diabetes has taken on significant importance in the fight against non-communicable diseases. Glycosylated hemoglobin (HbA1c) is an indicator of long-term (over the past 2 to 3 months) glucose metabolism [10, 11]. Compared to other measures used to measure glucose metabolism, HbA1c is less variable in the body, has greater pre-analytical stability, and has fewer daily disruptions during stress, dietary changes, or disease [12]. Moreover, HbA1c is the gold standard for assessing blood sugar control in people with diabetes and is linked to the risk of long-term complications [13]. Environmental contaminants may have a substantial impact on the development of diabetes, even if obesity, sedentary lifestyles, and poor eating habits are well-known risk factors for the disease [14–18].

Previous systematic reviews, based on abundant epidemiological studies and in vivo and in vitro studies, have raised potential health concerns about the widespread and extensive usage of glyphosate [4, 19, 20]. Even if opinions on the potential carcinogenicity, endocrine disrupting effects and other health impacts of glyphosate are still debatable [20], further validation is needed. Currently, research on whether glyphosate levels affect glucose metabolism is sparse. We hypothesized that glyphosate concentrations may be associated with HBA1c levels and diabetes prevalence. Here, we intend 1) to assess the association between urinary glyphosate levels and HbA1c levels, and 2) to evaluate the association between urinary glyphosate levels and the prevalence of diabetes based on the NHANES.

## Method

### Study population

**Fig. 1:**
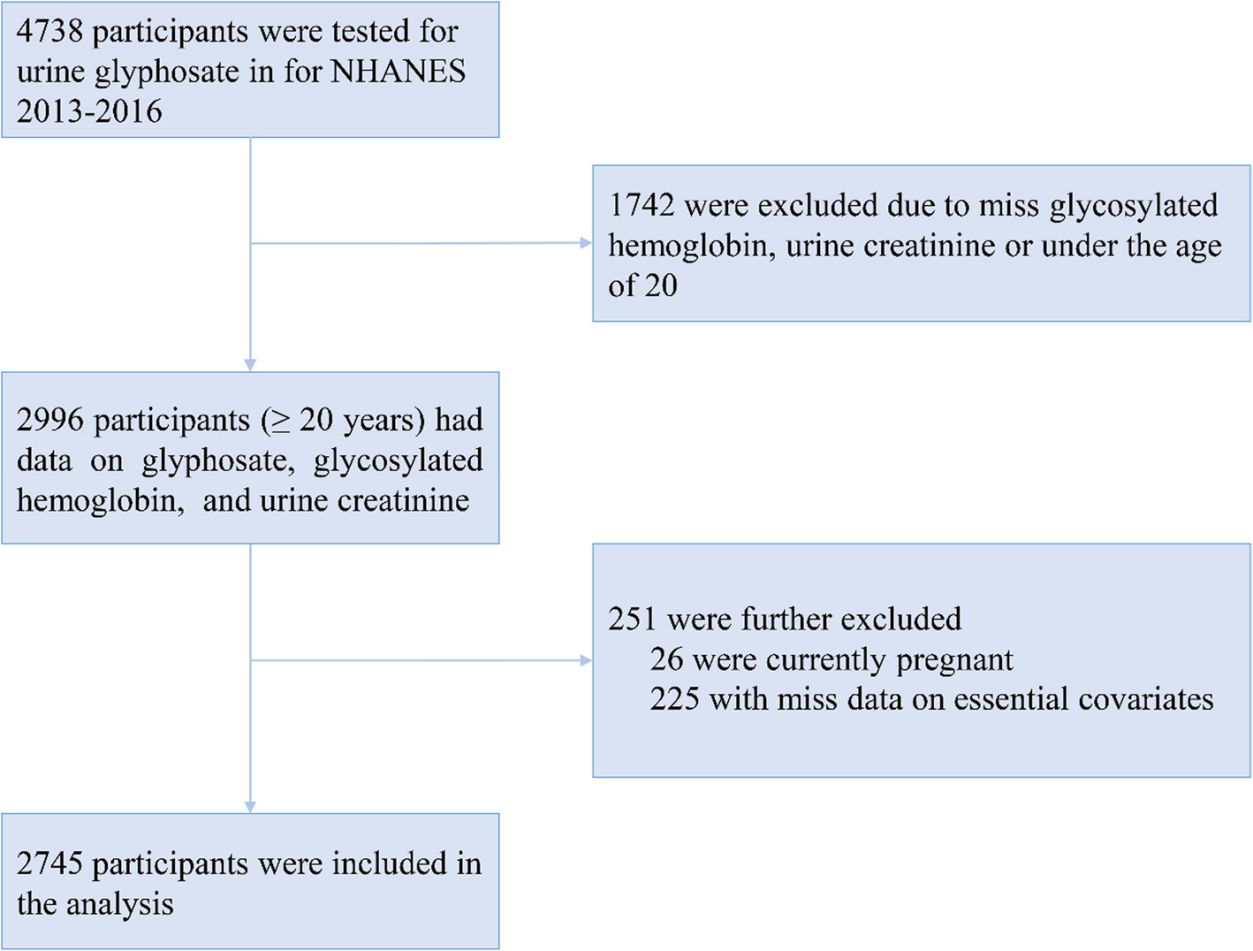
The flow chart of participants selection

The NHANES is a nationwide survey intended to evaluate the health and nutritional status of the U.S. civilian population [21]. The Research Ethics Review Board of the National Center for Health Statistics approved the study protocol. Each participant in the study provided their agreement in writing to take part in the NHANES program. This study included 4738 participants who had urinary glyphosate levels tested in the NHANES during 2013–2016. There were 2,996 participants (≥ 20 years) with data on urinary glyphosate concentration, glycosylated hemoglobin (HbA1c), and urinary creatinine. Additionally, participants who were pregnant (*n* = 26), or who missed covariate information (*n* = 225) including body mass index (BMI), alcohol consumption, or serum cotinine levels were excluded. The remaining 2,745 participants were included in the final analysis (Fig. 1).

### Definitions of diabetes

The diabetes mellitus patients included in this study were all type I, type II, etc. The following criteria were used to define diabetes: self-reported diabetes, current use of hypoglycemic agents, self-reported current use of insulin, or an HbA1c level ≥ 6.5% [22]. The relevant self-reported assessments are described in detail in the NHANES document [23, 24].

### Urinary glyphosate concentrations

The glyphosate levels of the study subjects were measured by the spot urine they provided, which was previously stored at -70 °C for testing. The samples (200 μL) were analyzed by 2D-on-line ion chromatography coupled with tandem mass spectrometry (IC- MS/MS). The detailed expatiation of laboratory procedures is available in previous research [25]. The limit of detection (LOD) was 0.2 μg/L for urinary glyphosate level. For concentrations below the LOD, NHANES reported a value (LOD/√2). The level of glyphosate in the urine was calibrated using urinary creatinine and then expressed as μg/g Cr to take the dilution of the urine into consideration.

### Covariates

Covariates were choosen based on biological consideration and previous literature [15, 22]. These covariates include age (< 40, 40–59, ≥ 60 years old), sex, race, BMI (< 25, 25–29.9, and ≥ 30 kg/m^2^), smoking status (Smoking was defined as serum cotinine level ≥ 10 ng/ml or having smoked at least 100 cigarettes in life), alcohol consumption (< 12 and ≥ 12 drinks/year), education levels (under high school, high school graduate, above high school), family poverty income ratio (PIR) (< 1 and ≥ 1) [26], physical activity (< 600, ≥ 600 MET-minutes/week) [27], and survey cycles of NHANES (2013–2014 and 2015–2016).

### Statistical analysis

Descriptive analyses were conducted for the characteristics of the participants and laboratory measurements of urinary glyphosate levels and HbA1c. The relevant characteristics of the subjects with and without diabetes were compared using the non-parametric Mann‒Whitney U test and the chi-square test.

The urinary glyphosate concentration data were skewed and natural logarithmic transformations were performed before statistical analysis. Generalized linear models (GLMs) were applied to explore the relationships between urinary glyphosate concentrations and HbA1c levels and the prevalence of diabetes. The results of the linear regression were converted and presented as the percent change. For each doubling of urinary glyphosate concentrations, the formula of (e ^(ln2× β)^ -1) × 100% was applied to calculate the percentage change in HbA1c [28]. Moreover, GLMs were constructed by dividing the urinary glyphosate levels into quartiles and choosing the lowest quartile as the baseline. Continuous variables representing the median value for each quartile were utilized to analyze the linear trend in the model [29]. The formula of (e ^β^- 1) × 100% was applied to calculate the percentage change in HbA1c related to quartiles of urinary glyphosate level [30]. To explore the dose–response relationship of urinary glyphosate concentrations with HbA1c levels and the prevalence of diabetes, restricted cubic splines (RCSs) [31] were applied with 3 knots (located at the 10th, 50th, and 90th percentiles of the natural log-transformed urinary glyphosate levels). The reference level was set at the 25th percentile of the natural log-transformed urinary glyphosate levels. The covariates in all analyses included sex, age, race, BMI, family PIR, smoking status, alcohol consumption, education levels, physical activity, and the survey cycle of the NHANES.

Stratification analysis and sensitivity analysis were performed to ensure the reliability of the results and explore the potential impact of other factors on the relationship of urinary glyphosate concentrations with HbA1c levels and the prevalence of diabetes. Subgroup analysis was conducted stratified by physical activity, sex, BMI (< 30 and ≥ 30 kg/m^2^) [32], and smoking status. Cross-product terms are added to the GLMs to evaluate the interaction effects. To examine the interaction between subgroups, the Wald test was conducted. Moreover, the relationships of urinary glyphosate concentrations with HbA1c levels and the prevalence of diabetes were evaluated by excluding subjects aged ≥ 75 years [14] or with abnormal glyphosate levels (higher than the upper quartile by three times the interquartile range (IQR) or less than the lower quartile by three times the IQR) or by additionally adjusting for carbohydrate intake.

All the statistical analyses were conducted using R (version 4.2.2), and a two-sided *p* value < 0.05 was considered statistically significant.

## Results

### Characteristics of the study population

A total of 2745 participants were included in the study, and the prevalence of diabetes was 16.4%. All demographic characteristics are presented in Table 1. The diabetic group was older and had a greater BMI than the non-diabetic group (*P* < 0.001). Participants with diabetes were more likely to be less educated and inactive, and to be more impoverished than those without diabetes (*P* < 0.001). The majority of the population in the survey were non-Hispanic white, consumed alcohol, and smoked.

**Table 1:** Baseline characteristics of the study participants

Characteristics	Total	Diabetic	Non-diabetic	*P* value
	(*N* = 2745)	(*N* = 450)	(*N* = 2295)	
Age				< 0.001
< 40	861 (31.4%)	33 (7.3%)	828 (36.1%)	
40–59	959 (34.9%)	152 (33.8%)	807 (35.2%)	
≥ 60	925 (33.7%)	265 (58.9%)	660 (28.7%)	
Sex				0.372
Male	1341 (48.9%)	229 (50.9%)	1112 (48.5%)	
Female	1404 (51.1%)	221 (49.1%)	1183 (51.5%)	
BMI (kg/m^2^)				< 0.001
< 25	775 (28.2%)	47 (10.4%)	728 (31.7%)	
25–29.9	859 (31.3%)	126 (28%)	733 (32.0%)	
≥ 30	1111 (40.5%)	277 (61.6%)	834 (36.3%)	
Race				< 0.001
Mexican American	428 (15.6%)	89 (19.8%)	339 (14.8%)	
Other Hispanic	299 (10.9%)	57 (12.7%)	242 (10.5%)	
Non-Hispanic White	1132 (41.2%)	141 (31.3%)	991 (43.2%)	
Non-Hispanic Black	521 (19.0%)	108 (24%)	413 (18.0%)	
Other race	365 (13.3%)	55 (12.2%)	310 (13.5%)	
Family PIR				< 0.001
< 1	570 (20.8%)	128 (28.4%)	442 (19.3%)	
≥ 1	2175 (79.2%)	322 (71.6%)	1853 (80.7%)	
Education				< 0.001
Under high school	565 (20.6%)	135 (30.0%)	430 (18.7%)	
High school or equivalent	615 (22.4%)	100 (22.2%)	515 (22.5%)	
Above high school	1565 (57.0%)	215 (47.8%)	1350 (58.8%)	
Smoking				0.190
Yes	1283 (46.7%)	223 (49.6%)	1060 (46.2%)	
No	1462 (53.3%)	227 (50.4%)	1235 (53.8%)	
Alcohol consumption (drinks/year)				< 0.001
< 12	800 (29.1%)	170 (37.8%)	630 (27.5%)	
≥ 12	1945 (70.9%)	280 (62.2%)	1665 (72.5%)	
Physical activity				< 0.001
Inactive	1093 (39.8%)	234 (52.0%)	859 (37.4%)	
Active	1652 (60.2%)	216 (48.0%)	1436 (62.6%)	
Survey cycle				0.022
2013–2014	1456 (53.0%)	216 (48.0%)	1240 (54.0%)	
2015–2016	1289 (47.0%)	234 (52.0%)	1055 (46.0%)	

*Abbreviations*: *BMI* body mass index, *Family PIR* Family income to poverty ratio

### Distribution of urinary glyphosate concentrations and HbA1c levels

The levels of urinary glyphosate of the study subjects are demonstrated in Table S1. The detection rate for urinary glyphosate was 74.1%. The median and geometric mean of the corrected urinary glyphosate levels in this study were 0.376 and 0.388 μg/g creatinine, respectively, while the median and geometric mean of the uncorrected urinary glyphosate levels were 0.357 and 0.372 μg/L, respectively. Urinary glyphosate concentrations (creatinine-corrected and uncorrected) were significantly higher in study subjects with diabetes than in those without diabetes (*P* < 0.001). The distributions of HbA1c are shown in Table S2. The geometric mean (GM) of HBA1c levels in the general population, diabetic population and non-diabetic population were 5.72, 7.39 and 5.44, respectively.

### Associations between urinary glyphosate levels and HbA1c

**Fig. 2:**
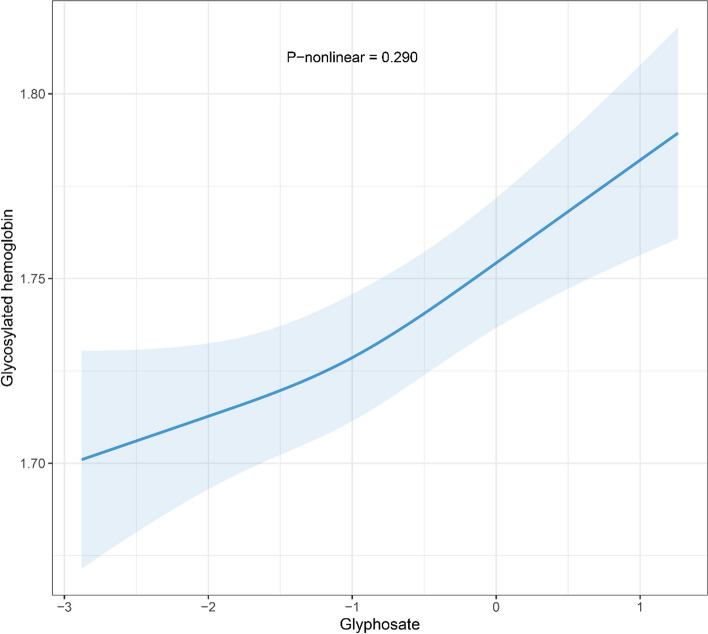
Restricted cubic splines for the relationship between the ln-transformed concentrations of urinary glyphosate and ln-transformed glycosylated hemoglobin. Model was adjusted for age, sex, body mass index, race, family income to poverty ratio, education, smoking status, drinking, physical activity, and survey cycle

The associations of urinary glyphosate levels and HbA1c are presented in Table 2. After adjusting for covariates, per doubling of the urinary glyphosate concentration was associated with a 1.49% (95% CI: 0.99, 2.00; *P* < 0.001) increase in the HbA1c level. Compared with the lowest quartile of glyphosate, the third glyphosate quartile was associated with a 2.37% (95% CI: 0.79, 3.97; *P* = 0.003) increase in the HbA1c level, and the highest glyphosate quartile was associated with a 4.19% (95% CI: 2.54, 5.85; *P* < 0.001) increase in the HbA1c level. The linear trend test showed a significant positive linear relationship between the urinary glyphosate concentrations and the HbA1c levels (*P* for trend < 0.001). According to the RCS model, the results showed a significant linear dose‒response relationship between glyphosate levels and HbA1c (overall association *P* < 0.001; *P* = 0.290 for nonlinearity) (Fig. 2).

**Table 2:** Associations of urinary glyphosate levels with the glycosylated hemoglobin

Urinary glyphosate (μg/g creatinine)	Unadjusted percentage change (95% CI)	*P* value	Adjusted percentage change (95% CI)	*P* value
Continuous	1.90 (1.37, 2.43)	< 0.001	1.49 (0.99, 2.00)	< 0.001
Quartiles				
Q1 (< 0.224)	0 (reference)		0 (reference)	
Q2 (0.224–0.376)	1.39 (-0.30, 3.11)	0.107	1.02 (-0.51, 2.57)	0.191
Q3 (0.376–0.671)	3.06 (1.34, 4.80)	< 0.001	2.37 (0.79, 3.97)	0.003
Q4 (≥ 0.671)	5.30 (3.54, 7.08)	< 0.001	4.19 (2.54, 5.85)	< 0.001
*P* for trend	< 0.001		< 0.001	

Adjusted for age, sex, body mass index, race, family income to poverty ratio, education, smoking status, drinking, physical activity, and survey cycle

*P* for trend across quartiles of urinary glyphosate levels

Abbreviation: *CI* confidence intervals

Subgroup analyses were performed to estimate the effects of sex, BMI, smoking status, and physical activity on the association between glyphosate and HbA1c. A significant positive association between urinary glyphosate concentrations and HbA1c levels was found in all the subgroups (Table S3, Table S4, Table S5, Table S6).

### Associations between urinary glyphosate levels and the diabetes prevalence

**Fig. 3:**
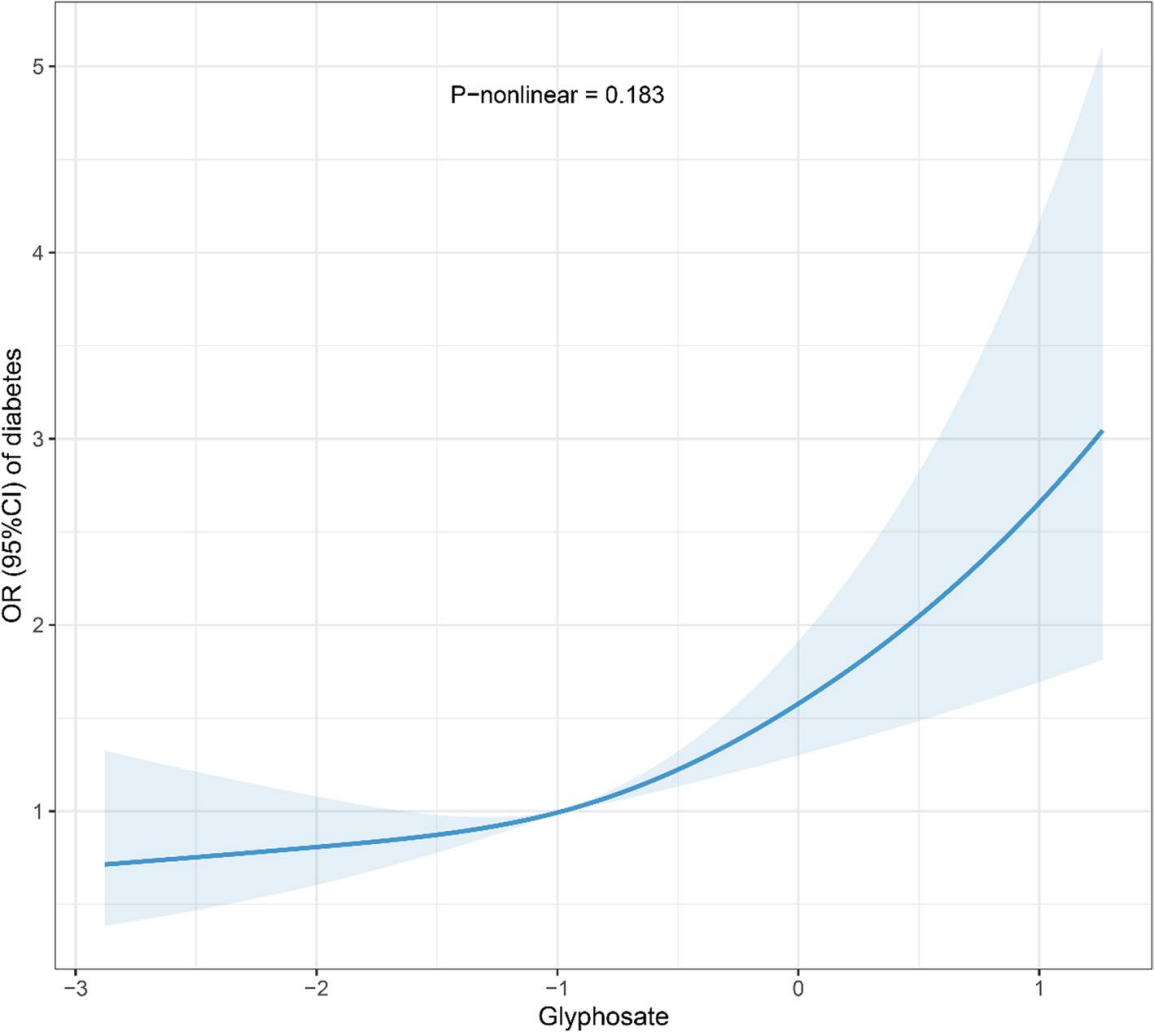
Restricted cubic splines for the relationship between the ln-transformed concentrations of urinary glyphosate and the prevalence of diabetes. Model was adjusted for age, sex, body mass index, race, family income to poverty ratio, education, smoking status, drinking, physical activity, and survey cycle

The associations of urinary glyphosate levels and the diabetes prevalence are presented in Table 3. After adjusting for covariates, the urinary glyphosate concentration was significantly associated with an increased prevalence of diabetes of 46% (95% CI: 1.26, 1.69; *P* < 0.001). According to the categorical model, a significant positive correlation between urinary glyphosate concentration and the prevalence of diabetes mellitus was still demonstrated (*P* for trend < 0.001), and the prevalence of diabetes was elevated 1.89-fold (95% CI: 1.37, 2.63; *P* < 0.001) in the highest quartile of glyphosate compared with the first quartile of glyphosate. The RCS was also used to analyze the nonlinear correlation between ln-transformed glyphosate concentrations and the prevalence of diabetes (Fig. 3). The results revealed a significant linear dose‒response relationship of ln-transformed glyphosate levels with the prevalence of diabetes (overall association *P* < 0.001; *P* = 0.183 for nonlinearity).

**Table 3:** Associations of urinary glyphosate levels with the prevalence of diabetes

Urinary glyphosate	Unadjusted OR (95% CI)	*P* value	Adjusted OR (95% CI)	*P* value
Continuous	1.52 (1.33, 1.73)	< 0.001	1.46 (1.26, 1.69)	< 0.001
Quartiles				
Q1 (< 0.224)	1 (reference)		1 (reference)	
Q2 (0.224–0.376)	1.09 (0.80, 1.50)	0.582	1.01 (0.72, 1.41)	0.977
Q3 (0.376–0.671)	1.37 (1.02, 1.87)	0.038	1.29 (0.90, 1.74)	0.191
Q4 (≥ 0.671)	2.08 (1.57, 2.79)	< 0.001	1.89 (1.37, 2.63)	< 0.001
*P* for trend	< 0.001		< 0.001	

Adjusted for age, sex, body mass index, race, family income to poverty ratio, education, smiking status, drinking, physical activity, and survey cycle

*P* for trend across quartiles of urinary glyphosate levels

*Abbreviation*: *CI* confidence intervals

Subgroup analysis was performed to further estimate the influence of sex, BMI, smoking status, and physical activity on the relationship of glyphosate with the prevalence of diabetes. Glyphosate was positively associated with the prevalence of diabetes in all subgroups (Table S3, Table S4, Table S5, Table S6).

### Sensitivity analyses

In the sensitivity analyses by excluding the older subjects (≥ 75 years old), or outliers of urinary glyphosate concentrations, or by additionally adjusting for carbohydrate intake, significant positive associations were observed of urinary glyphosate concentrations with HbA1c levels and the prevalence of diabetes (Table S7, Table S8 and Table S10), consistent with previous results. In addition, after excluding both older subjects (≥ 75 years old) and urinary glyphosate concentration outliers, the results still showed a significant positive association of urinary glyphosate levels with HbA1c (percent change: 1.68; 95% CI: 1.10, 2.26) and the prevalence of diabetes (OR: 1.51; 95% CI: 1.26, 1.80) (Table S9).

## Discussion

The findings of our study indicated that the urinary glyphosate concentrations were positively correlated with HbA1c levels and the prevalence of diabetes after adjusting for covariates. In all the subgroups, there was still a significant positive association between urinary glyphosate levels and HbA1c levels and prevalence of diabetes mellitus.

Glyphosate exposure in humans occurs via skin contact, inhalation, and dietary exposure [5, 6]. With a short elimination half-life( 5.5–10 h) [33], previous investigations have demonstrated that only a small fraction (1–6%) of ingested glyphosate is promptly removed in urine as an unaltered compound [34, 35]. However, most studies have assessed human exposure to glyphosate by measuring glyphosate levels in urine [2, 36, 37]. Due to the widespread use of urinary glyphosate levels, it is comparable across populations by assessing urinary glyphosate levels. The concentrations of urinary glyphosate in the present study were similar to those in the Agricultural Health Study from the United States [2] and a survey in Mexico [38], and higher than those in a Swedish study [35]. In addition, the glyphosate concentrations in this study were lower than exposure levels in adults (Mean = 0.87 μg/L) in an Irish study [33]. Despite only a small portion of ingested glyphosate is rapidly cleared from the urine as unchanged compounds, the detection rates of urinary glyphosate of the populations in these studies was high.

Despite extensive research and public interest in the carcinogenic effects of glyphosate, epidemiological evidence on other health effects in humans remains limited. Currently, epidemiological studies suggest that exposure to glyphosate may affect human sex hormones [39], gestational length [37, 40], DNA methylation [41], lung function [42] and biomarkers of oxidative stress [2]. This is the first time, as far as we are aware, that the relationship of glyphosate concentrations with HbA1c levels has been examined. Moreover, urinary glyphosate concentrations were shown to be associated with elevated HbA1c levels and increased diabetes prevalence, which supports the hypothesis that glyphosate may have biological effects in humans. However, the effects of sustained low glyphosate exposure on human health are still unknown due to the lack of monitoring research.

Due to the current lack of epidemiological studies in this area, comparing our findings to those of other studies is difficult. According to previous research, glyphosate plays a key role in metabolic pathways in mammals [43]. Earlier studies on animals have shown that exposure to glyphosate was implicated in elevated blood glucose [44–46] and HbA1c [47]. In addition, previous animal studies have found that exposure to glyphosate can lead to insulin resistance [46, 47]. This study extends the limited evidence on the health effects of exposure to glyphosate based on internal-exposure assessments.

From earlier investigations of glucose metabolism and insulin resistance, it is possible to deduce several probable mechanisms via which glyphosate may affect the onset of diabetes. Oxidative stress caused by reactive oxygen species in living systems has both positive and negative consequences [48], contributing to diabetogenesis and the emergence of problems related to diabetes [49, 50]. Glyphosate can cause oxidative stress [51], which plays a significant role in pancreatic β-cell function and survival. Oxidative stress induces the activation of AMP-activated protein kinase, the inhibition of mammalian target of rapamycin, and the activation of c-Jun N-terminal kinase in pancreatic β cells [49]. In addition, oxidative stress can cause hyperglycemia by stimulating the sympathetic nervous system and the hypothalamic–pituitary–adrenal axis, leading to insulin resistance [46]. Moreover, exposure to glyphosate can cause an inflammatory response [46], which may have an impact on the development of diabetes by producing insulin resistance and then exacerbated in the case of high blood sugar, thereby increasing the long-term consequences of diabetes [52]. Exposure to glyphosate also induces skeletal muscle insulin resistance by modulating IRS-1/PI3K/Akt insulin signaling molecules, leading to the development of T2DM [47]. Finally, glyphosate may be an endocrine disrupting substance with endocrine disrupting effects [20]. Hyperinsulinemia can be caused by the overstimulation of estrogen receptors in the β cells of the pancreas. Subsequently, pancreatic cells, liver, and muscle develop insulin resistance due to elevated insulin signaling. [53]. In conclusion, glyphosate may contribute to the development of diabetes through a variety of routes, while the application of epidemiological evidence to low levels of persistent exposure in the general public may be restricted by experimental findings based on high levels of exposure.

Several strengths of this study are notable. First, our study described urinary glyphosate concentrations in adults with a relatively large sample size and is the first to evaluate the relationship of urinary glyphosate concentrations with HbA1c levels in a population. Second, glyphosate concentrations are measured by internal exposure, making the assessment more accurate. Moreover, compared with other blood glucose indicators, HbA1c is not susceptible to short-term fluctuations in other factors and has better stability. Finally, subgroup analysis and sensitivity analysis were performed to explore the potential impact of relevant factors on the study findings.

It is also crucial to be aware of the potential limitations of this study. First, it is not possible to establish a temporal causal association between glyphosate concentrations and the risk of diabetes due to the cross-sectional design of the NHANES. Second, since glyphosate has a short half-life [34], the amount measured of glyphosate at one time point may not accurately reflect long-term exposure. Thus, it may be advantageous for future studies to evaluate exposure over a range of time. Finally, the exposure levels of aminomethylphosphonic acid, the primary metabolite of glyphosate, were not evaluated.

## Conclusions

In conclusion, our findings indicate that the urinary glyphosate concentration is positively related to HBA1c levels and the prevalence of diabetes in adults. Our findings need to be confirmed, potential biological mechanisms by which glyphosate affects glycemic homeostasis should be explored, and the relationship of glyphosate levels over time with the development of diabetes should be further examined.

### Supplementary Information

Supplementary Material 1.

## Data Availability

The datasets of National Health and Nutrition Examination Survey are available at https://www.cdc.gov/nchs/nhanes/index.htm.

## Abbreviations

NHANES: National Health and Nutrition Examination Survey
RCS: Restricted cubic spline
HbA1c: Glycosylated hemoglobin
GLMs: Generalized linear models
LOD: Limit of detection
BMI: Body mass index
IQR: Interquartile range
PIR: Poverty income ratio
GM: Geometric mean
