# Supplementary material for: Relationship of urinary glyphosate concentrations with glycosylated hemoglobin and diabetes in US adults: a cross-sectional study
Source: BMC Public Health. 2024 Jun 20;24:1644. doi: 10.1186/s12889-024-19126-0 (PMC11188266; doi:10.1186/s12889-024-19126-0)
Supplement: Supplementary file 1 — Supplementary Material 1. [file 12889_2024_19126_MOESM1_ESM.docx]

**Relationship of urinary glyphosate concentration with glycosylated hemoglobin and diabetes in US adults: A cross-sectional study**

**Table of Contents:**

**Table S1:** Distributions of maternal urinary glyphosate levels.

**Table S2:** Glycosylated hemoglobin in the participants of this study.

**Table S3:** Associations of urinary glyphosate concentrations with glycosylated hemoglobin and the prevalence of diabetes, stratified by sex.

**Table S4:** Associations of urinary glyphosate concentrations with glycosylated hemoglobin and the prevalence of diabetes, stratified by body mass index.

**Table S5:** Associations of urinary glyphosate concentrations with glycosylated hemoglobin and the prevalence of diabetes, stratified by physical activity.

**Table S6:** Associations of urinary glyphosate concentrations with glycosylated hemoglobin and the prevalence of diabetes, stratified by smoking status.

**Table S7:** Associations of urinary glyphosate concentrations with the prevalence of diabetes and glycosylated hemoglobin by excluding the older subjects (≥ 75 years).

**Table S8:** Associations of urinary glyphosate concentrations with the prevalence of diabetes and glycosylated hemoglobin by excluding the subjects with abnormal glyphosate levels.

**Table S9:** Associations of urinary glyphosate concentrations with the prevalence of diabetes and glycosylated hemoglobin by excluding the subjects ≥ 75 years old and with abnormal glyphosate levels.

**Table S10:** Associations of urinary glyphosate concentrations with the prevalence of diabetes and glycosylated hemoglobin level additional adjustment for carbohydrate intake.

| **Table S1** Distributions of urinary glyphosate levels. | | | | | | | | | |
| --- | --- | --- | --- | --- | --- | --- | --- | --- | --- |
| Urinary glyphosate levels | Total (n = 2,745) | | |  | Diabetic (N = 450) |  | Non-diabetic (N = 2,295) |  | *P*-value |
|  | LOD (ug/L) | GM | Median (IQR) |  | Median (IQR) |  | Median (IQR) |  |  |
| Uncorrected glyphosate (ug/L) | 0.2 | 0.372 | 0.357 (0.141, 0.616) |  | 0.428 (0.269, 0.780) |  | 0.344 (0.141, 0.589) |  | < 0.001 |
| Creatinine-corrected glyphosate (μg/g creatinine) |  | 0388 | 0.376 (0.224, 0.671) |  | 0.486 (0.262, 0.885) |  | 0.362 (0.220, 0.628) |  | < 0.001 |
| Abbreviation: LOD; limit of detection; GM, geometric mean; IQR: interquartile range. | | | | | | | | | |

| **Table S2** Glycosylated hemoglobin in the participants of this study. | | | | | | |
| --- | --- | --- | --- | --- | --- | --- |
| Glycosylated hemoglobin (%) | GM | Percentile | | | | |
|  |  | 5th | 25th | 50th | 75th | 95th |
| Total (N = 2,745) | 5.72 | 4.8 | 5.2 | 5.5 | 5.9 | 7.9 |
| Diabetic (N = 450) | 7.39 | 5.6 | 6.4 | 7.0 | 8.3 | 11.6 |
| Non-diabetic (N = 2,295) | 5.44 | 4.8 | 5.2 | 5.5 | 5.7 | 6.1 |
| *P* value | < 0.001 |  |  |  |  |  |
| Abbreviations: GM, geometric mean. | | | | | | |

| **Table S3** Associations of urinary glyphosate concentrations with glycosylated hemoglobin and the prevalence of diabetes, stratified by sex. | | | | | | |
| --- | --- | --- | --- | --- | --- | --- |
| Urinary glyphosate | Percentage change (95% CI) | | *P* interaction | OR (95%CI) | | *P* interaction |
|  | Male | Female |  | Male | Female |  |
| Continuous | 1.56 (0.83, 2.29) | 1.45 (0.74, 2.16) | 0.701 | 1.42 (1.15, 1.74) | 1.51 (1.22, 1.88) | 0.980 |
| Quartiles |  |  | 0.161 |  |  | 0.170 |
| Q1 (< 0.224) | 0 (reference) | 0 (reference) |  | 1 (reference) | 1 (reference) |  |
| Q2 (0.224–0.376) | 1.17 (-0.92, 3.29) | 0.66 (-1.60, 2.98) |  | 0.98 (0.61, 1.56) | 1.01 (0.61, 1.69) |  |
| Q3 (0.376–0.671) | 3.95 (1.70, 6.23) | 0.92 (-1.32, 3.21) |  | 1.69 (1.07, 2.68) | 0.97 (0.59, 1.60) |  |
| Q4 (≥ 0.671) | 4.05 (1.69, 6.46) | 4.01 (1.67, 6.39) |  | 1.86 (1.18, 2.94) | 1.86 (1.16, 3.04) |  |
| *P* for trend | < 0.001 | < 0.001 |  | 0.002 | < 0.001 |  |
| Abbreviations: OR: odds ratio; CI: confidence intervals.  Models were adjusted for age, body mass index, race, family income to poverty ratio, education, smoking status, drinking, physical activity, and survey cycle.  *P* for trend across quartiles of urinary glyphosate level. | | | | | | |

| **Table S4** Associations of urinary glyphosate concentrations with glycosylated hemoglobin and the prevalence of diabetes, stratified by body mass index. | | | | | | |
| --- | --- | --- | --- | --- | --- | --- |
| Urinary glyphosate | Percentage change (95% CI) | | *P* interaction | OR (95%CI) | | *P* interaction |
|  | BMI < 30 kg/m^2^ | BMI ≥ 30 kg/m^2^ |  | BMI < 30 kg/m^2^ | BMI ≥ 30 kg/m^2^ |  |
| Continuous | 1.07 (0.51, 1.64) | 1.98 (1.04, 2.94) | 0.026 | 1.43 (1.15, 1.78) | 1.43 (1.17, 1.75) | 0.940 |
| Quartiles |  |  | 0.142 |  |  | 0.475 |
| Q1 (< 0.224) | 0 (reference) | 0 (reference) |  | 1 (reference) | 1 (reference) |  |
| Q2 (0.224–0.376) | 1.30 (-0.47, 3.10) | 0.33 (-2.40, 3.14) |  | 1.13 (0.65, 1.97) | 0.94 (0.61, 1.44) |  |
| Q3 (0.376–0.671) | 2.61 (0.79, 4.48) | 1.92 (-0.89, 4.81) |  | 1.63 (0.97, 2.78) | 1.04 (0.67, 1.60) |  |
| Q4 (≥ 0.671) | 3.21 (1.39, 3.07) | 5.13 (2.01, 8.33) |  | 1.92 (1.17, 3.21) | 1.82 (1.18, 2.81) |  |
| *P* for trend | < 0.001 | < 0.001 |  | 0.005 | < 0.001 |  |
| Abbreviations: OR: odds ratio; CI: confidence intervals.  Models were adjusted for age, sex, race, family income to poverty ratio, education, smoking status, drinking, physical activity, and survey cycle.  *P* for trend across quartiles of urinary glyphosate level. | | | | | | |

| **Table S5** Associations of urinary glyphosate concentrations with glycosylated hemoglobin and the prevalence of diabetes, stratified by physical activity. | | | | | | |
| --- | --- | --- | --- | --- | --- | --- |
| Urinary glyphosate | Percentage change (95% CI) | | *P* interaction | OR (95%CI) | | *P* interaction |
|  | Inactive | Active |  | Inactive | Active |  |
| Continuous | 1.77 (0.89, 2.65) | 1.36 (0.76, 1.97) | 0.282 | 1.62 (1.34, 1.97) | 1.34 (1.08, 1.65) | 0.353 |
| Quartiles |  |  | 0.167 |  |  | 0.163 |
| Q1 (< 0.224) | 0 (reference) | 0 (reference) |  | 1 (reference) | 1 (reference) |  |
| Q2 (0.224–0.376) | 0.52 (-2.32, 3.37) | 1.25 (-0.54, 3.07) |  | 0.92 (0.58, 1.47) | 1.19 (0.75, 1.89) |  |
| Q3 (0.376–0.671) | 1.03 (-1.81, 3.95) | 3.27 (1.44, 5.14) |  | 1.38 (0.89, 2.16) | 1.34 (0.85, 2.13) |  |
| Q4 (≥ 0.671) | 4.96 (2.08, 7.91) | 3.51 (1.56, 5.51) |  | 2.24 (1.47, 3.46) | 1.56 (0.98, 2.51) |  |
| *P* for trend | < 0.001 | < 0.001 |  | < 0.001 | 0.064 |  |
| Abbreviations: OR: odds ratio; CI: confidence intervals.  Models were adjusted for age, sex, race, family income to poverty ratio, education, smoking status, drinking, body mass index, and survey cycle.  *P* for trend across quartiles of urinary glyphosate level. | | | | | | |

| **Table S6** Associations of urinary glyphosate concentrations with glycosylated hemoglobin and the prevalence of diabetes, stratified by smoking. | | | | | | |
| --- | --- | --- | --- | --- | --- | --- |
| Urinary glyphosate | Percentage change (95% CI) | | *P* interaction | OR (95%CI) | | *P* interaction |
|  | No-smoking | Smoking |  | No-smoking | Smoking |  |
| Continuous | 1.29 (0.60, 1.98) | 1.72 (0.98, 2.48) | 0.337 | 1.46 (1.19, 1.81) | 1.43 (1.16, 1.77) | 0.909 |
| Quartiles |  |  | 0.537 |  |  | 0.555 |
| Q1 (< 0.224) | 0 (reference) | 0 (reference) |  | 1 (reference) | 1 (reference) |  |
| Q2 (0.224–0.376) | 0.83 (-1.28, 2.98) | 1.38 (-0.87, 3.67) |  | 0.99 (0.60, 1.63) | 1..03 (0.64, 1.64) |  |
| Q3 (0.376–0.671) | 2.17 (0.01, 4.37) | 2.62 (0.31, 4.98) |  | 1.48 (0.92, 2.39) | 1.04 (0.65, 1.67) |  |
| Q4 (≥ 0.671) | 3.17 (0.94, 5.43) | 5.49 (3.02, 8.02) |  | 1.82 (1.14, 2.95) | 1.95 (1.24, 3.09) |  |
| *P* for trend | 0.004 | < 0.001 |  | 0.003 | < 0.001 |  |
| Abbreviations: OR: odds ratio; CI: confidence intervals.  Models were adjusted for age, sex, race, family income to poverty ratio, body mass index, education, physical activity, drinking, and survey cycle.  *P* for trend across quartiles of urinary glyphosate level. | | | | | | |

| **Table S7** Associations of urinary glyphosate concentrations with the prevalence of diabetes and glycosylated hemoglobin by excluding the older subjects (≥ 75 years). | | | |
| --- | --- | --- | --- |
| Urinary glyphosate | N | OR (95% CI) | Percentage change (95% CI) |
| Continuous | 2475 | 1.46 (1.25, 1.72) | 1.54 (1.00, 2.09) |
| Quartiles |  |  |  |
| Q1 (< 0.217) | 621 | 1 (reference) | 0 (reference) |
| Q2 (0.217–0.362) | 618 | 1.10 (0.76, 1.58) | 1.08 (-0.56, 2.74) |
| Q3 (0.362–0.635) | 617 | 1.33 (0.93, 1.90) | 2.21 (0.54, 3.90) |
| Q4 (≥ 0.635) | 619 | 1.91 (1.35, 2.72) | 4.12 (2.38, 5.89) |
| *P* for trend |  | < 0.001 | < 0.001 |
| Abbreviations: OR: odds ratio; CI: confidence intervals.  Models were adjusted for age, sex, body mass index, race, family income to poverty ratio, education, smoking status, drinking, physical activity, and survey cycle.  *P* for trend across quartiles of urinary glyphosate level. | | | |

| **Table S8** Associations of urinary glyphosate concentrations with the prevalence of diabetes and glycosylated hemoglobin by excluding the subjects with abnormal glyphosate levels. | | | |
| --- | --- | --- | --- |
| Urinary glyphosate | N | OR (95% CI) | Percentage change (95% CI) |
| Continuous | 2687 | 1.52 (1.29, 1.80) | 1.64 (1.10, 2.19) |
| Quartiles |  |  |  |
| Q1 (< 0.222) | 672 | 1 (reference) | 0 (reference) |
| Q2 (0.222–0.368) | 671 | 0.94 (0.67, 1.33) | 0.93 (-0.61, 2.50) |
| Q3 (0.368–0.636) | 672 | 1.23 (0.88, 1.73) | 2.27 (0.68, 3.87) |
| Q4 (≥ 0.636) | 672 | 1.83 (1.32, 2.54) | 4.07 (2.42, 5.75) |
| *P* for trend |  | < 0.001 | < 0.001 |
| Abbreviations: OR: odds ratio; CI: confidence intervals.  Models were adjusted for age, sex, body mass index, race, family income to poverty ratio, education, smoking status, drinking, physical activity, and survey cycle.  *P* for trend across quartiles of urinary glyphosate level. | | | |

| **Table S9** Associations of urinary glyphosate concentrations with the prevalence of diabetes and glycosylated hemoglobin by excluding the subjects ≥ 75 years old and with abnormal glyphosate levels. | | | |
| --- | --- | --- | --- |
| Urinary glyphosate | N | OR (95% CI) | Percentage change (95% CI) |
| Continuous | 2429 | 1.51 (1.26, 1.80) | 1.68 (1.10, 2.26) |
| Quartiles |  |  |  |
| Q1 (< 0.216) | 672 | 1 (reference) | 0 (reference) |
| Q2 (0.216–0.355) | 671 | 1.05 (0.72, 1.51) | 0.84 (-0.80, 2.50) |
| Q3 (0.355–0.613) | 672 | 1.23 (0.86, 1.77) | 2.14 (0.47, 3.85) |
| Q4 (≥ 0.613) | 672 | 1.84 (1.29, 2.63) | 4.01 (2.26, 5.79) |
| *P* for trend |  | < 0.001 | < 0.001 |
| Abbreviations: OR: odds ratio; CI: confidence intervals.  Models were adjusted for age, sex, body mass index, race, family income to poverty ratio, education, smoking status, drinking, physical activity, and survey cycle.  *P* for trend across quartiles of urinary glyphosate level. | | | |

| **Table S10** Associations of urinary glyphosate concentrations with the prevalence of diabetes and glycosylated hemoglobin level additional adjustment for carbohydrate intake. | | | |
| --- | --- | --- | --- |
| Urinary glyphosate | N | OR (95% CI) | Percentage change (95% CI) |
| Continuous | 2745 | 1.47 (1.27, 1.70) | 1.50 (1.00, 2.01) |
| Quartiles |  |  |  |
| Q1 (< 0.224) | 686 | 1 (reference) | 0 (reference) |
| Q2 (0.224–0.376) | 687 | 1.02 (0.73, 1.44) | 1.03 (-0.50, 2.58) |
| Q3 (0.376–0.671) | 685 | 1.26 (0.90, 1.76) | 2.38 (0.80, 3.98) |
| Q4 (≥ 0.671) | 687 | 1.93 (1.39, 2.68) | 4.20 (2.55, 5.87) |
| *P* for trend |  | < 0.001 | < 0.001 |
| Abbreviations: OR: odds ratio; CI: confidence intervals.  Models were adjusted for age, sex, body mass index, race, family income to poverty ratio, education, smoking status, drinking, physical activity, carbohydrate intake, and survey cycle.  *P* for trend across quartiles of urinary glyphosate level. | | | |
